# Correction to: ISCEV Standard for clinical electro-oculography (2017 update)

**DOI:** 10.1007/s10633-018-9627-0

**Published:** 2018-03-14

**Authors:** Paul A. Constable, Michael Bach, Laura J. Frishman, Brett G. Jeffrey, Anthony G. Robson, for the International Society for Clinical Electrophysiology of Vision

**Affiliations:** 1 Discipline of Optometry and Vision Science, Flinders University, Adelaide, Australia; 2 Department of Ophthalmology, University Medical Center Freiburg, Freiburg, Germany; 3 College of Optometry, University of Houston, Houston, TX USA; 4 Ophthalmic Genetics and Visual Function Branch, National Eye Institute, Bethesda, MD USA; 5 Institute of Ophthalmology, University College London, London, UK; 6 Moorfields Eye Hospital, London, UK

## Correction to: Doc Ophthalmol (2017) 134:155 10.1007/s10633-017-9580-3

The article “ISCEV Standard for clinical electro-oculography (2017 update)”, written by Paul A. Constable, Michael Bach, Laura J. Frishman, Brett G. Jeffrey, Anthony G. Robson and for the International Society for Clinical Electrophysiology of Vision, was originally published Online First without open access. After publication in volume 134, issue 1, pp. 1–9 the authors decided to opt for Open Choice and to make the article an open access publication. Therefore, the copyright of the article has been changed to © The Author(s) 2017 and the article is forthwith distributed under the terms of the Creative Commons Attribution 4.0 International License (http://creativecommons.org/licenses/by/4.0/), which permits use, duplication, adaptation, distribution and reproduction in any medium or format, as long as you give appropriate credit to the original author(s) and the source, provide a link to the Creative Commons license, and indicate if changes were made.

The axis label in figure 5 should read 25 s/division and not 25 ms/division.

An erratum (10.1007/s10633-017-9580-3) was published to make the article open access. Unfortunately, the copyright of the article incorrectly remained © Springer-Verlag Berlin Heidelberg 2017. Therefore, the copyright of the article has been correctly changed to © The Author(s) 2018.

